# Vitamins, Coenzyme Q10, and Antioxidant Strategies to Improve Oocyte Quality in Women with Gynecological Cancers: A Comprehensive Review

**DOI:** 10.3390/antiox13121567

**Published:** 2024-12-19

**Authors:** Isaic Alexandru, Daciana Nistor, Alexandru Catalin Motofelea, Bianca-Astrid Cadar (Andone), Andreea Crintea, Carmen Tatu, Gheorghe Nicusor Pop, Andrei Nicolae Csep

**Affiliations:** 1 Department of General Surgery, “Victor Babes” University of Medicine and Pharmacy Timisoara, Eftimie Murgu Square 2, 300041 Timisoara, Romania; isaic.alexandru@umft.ro; 2 Department of Functional Sciences, Physiology, Centre of Imuno-Physiology and Biotechnologies (CIFBIOTEH), “Victor Babes” University of Medicine and Pharmacy, 300041 Timisoara, Romania; carmen.tatu@umft.ro; 3 Centre for Gene and Cellular Therapies in Cancer, 3000723 Timisoara, Romania; 4 Center for Molecular Research in Nephrology and Vascular Disease, Faculty of Medicine, “Victor Babes” University of Medicine and Pharmacy, 300041 Timisoara, Romania; alexandru.motofelea@umft.ro; 5 Interdisciplinary Research Institute in Bio-Nano-Sciences, Babes-Bolyai University, 42 T. Laurian Str., 400271 Cluj-Napoca, Romania; bianca.andone@ubbcluj.ro; 6 Faculty of Physics, Babes-Bolyai University, 1 M. Kogalniceanu Str., 400084 Cluj-Napoca, Romania; 7 Department of Molecular Sciences, University of Medicine and Pharmacy “Iuliu Hațieganu”, 400349 Cluj-Napoca, Romania; crintea.andreea@umfcluj.ro; 8 Center for Modeling Biological Systems and Data Analysis (CMSBAD), “Victor Babes” University of Medicine and Pharmacy, 300041 Timisoara, Romania; pop.nicusor@umft.ro; 9 Department of Psycho-Neuroscience and Recovery, Faculty of Medicine and Pharmacy, University of Oradea, 410087 Oradea, Romania; csep.andrei@uoradea.ro

**Keywords:** antioxidant, gynecological cancers, oocyte, fertility

## Abstract

Background: Gynecological cancers, including ovarian, cervical, and endometrial cancers, significantly affect both survival and reproductive health in women. Cancer treatments such as chemotherapy and radiotherapy can impair ovarian function, reducing oocyte quality and fertility potential. Objective: This review aims to evaluate how vitamins and antioxidants can enhance fertility and fertility preservation outcomes for women diagnosed with gynecological cancers, particularly in the context of assisted reproductive technologies (ART). Standard treatments for these cancers, including hysterectomy, bilateral salpingo-oophorectomy, radiation, and chemotherapy, often compromise ovarian function and oocyte quality. This review focuses on the potential role of these interventions in improving oocyte quality, thereby supporting successful fertility preservation and ART outcomes. Methods: A comprehensive narrative review of the current literature was conducted, examining the effects of vitamins A, C, D3, E, and Coenzyme Q10 on oocyte quality, particularly in the context of oxidative stress and inflammation induced by cancer and its treatments. Results: The evidence suggests that certain vitamins and antioxidants may mitigate oxidative damage and enhance oocyte quality. Vitamin A supports cumulus–oocyte complex integrity, while vitamins C and E act as potent antioxidants, reducing oxidative stress in ovarian tissues. Vitamin D3 enhances ovarian reserve markers and modulates inflammatory cytokines. Coenzyme Q10 improves mitochondrial function and reduces DNA damage, increasing oocyte viability and fertilization potential. Conclusions: The incorporation of specific vitamins and antioxidants into fertility preservation strategies may enhance oocyte quality in women with gynecological cancers. Although the preliminary findings are promising, further research is needed to determine optimal dosages and establish standardized protocols for clinical use.

## 1. Introduction

Gynecological cancers, including ovarian, cervical, and endometrial cancers, represent a substantial health burden for women globally. In 2020, it was estimated that gynecological cancers accounted for nearly 40% of all cancers diagnosed in women, contributing to 30% of all cancer-related mortality in this population [1,2]. Among these, cervical cancer is the most prevalent, with 604,127 new cases reported globally in 2020, and it remains a leading cause of cancer-related death, responsible for 3.4% of all cancer mortalities in women [2]. Endometrial cancer (corpus uteri) follows, with 417,367 new cases and a 1% contribution to cancer-related deaths, while ovarian cancer, although less common (313,959 new cases), exhibits a higher mortality rate of 2.1% [2]. Vulvar and vaginal cancers are rare, with 45,240 and 17,908 cases reported, respectively, but still contribute significantly to the overall cancer burden [2]. Beyond their impact on survival, these malignancies profoundly affect reproductive health. Both cancer-related genetic mutations and the adverse effects of oncological treatments (e.g., chemotherapy, radiotherapy) can compromise ovarian function and oocyte quality. Oocyte quality generally refers to the overall health and potential of an egg to be fertilized, develop into an embryo, and result in a successful pregnancy. Key indicators of oocyte quality include chromosome integrity, mitochondrial function, and cytoplasmic composition [3]. Chemotherapy, radiotherapy, and other factors can damage the genetic material and structural integrity of oocytes, leading to impaired fertility and diminished reproductive potential in affected women [4].

The increasing recognition of the importance of fertility preservation for women diagnosed with gynecological cancers has driven advancements in assisted reproductive technologies (ART). Techniques such as oocyte cryopreservation and in vitro fertilization (IVF) offer potential solutions for maintaining fertility, even when cancer treatments threaten ovarian reserve [5]. However, the success of these fertility preservation strategies largely depends on the initial quality of the oocytes before cryopreservation. Research indicates that the structural and functional integrity of oocytes is crucial for successful fertilization, embryo development, and pregnancy outcomes [6].

Emerging evidence suggests that certain nutritional interventions, including the use of vitamins and antioxidants, may play a key role in enhancing oocyte quality. These substances are believed to counteract oxidative stress and inflammation, which are exacerbated in the cancer microenvironment and further intensified by oncological treatments. Vitamins A, C, D3, E, and Coenzyme Q10 have shown potential in improving mitochondrial function, reducing oxidative damage, and stabilizing hormonal balance, all of which are critical factors for maintaining oocyte competency [7,8,9,10].

Given the increasing incidence of gynecological cancers and the growing demand for effective fertility preservation strategies, there is a need to explore the role of nutritional interventions in enhancing oocyte quality. This review aims to comprehensively examine the effects of vitamins and antioxidants on oocyte quality, with a focus on their potential application in fertility preservation for women diagnosed with gynecological cancers. By synthesizing current evidence, this review seeks to provide a clearer understanding of the mechanisms through which these substances may improve reproductive outcomes and to identify potential areas for future research.

## 2. Materials and Methods

### 2.1. Study Design

This manuscript is structured as a narrative review, focusing on the effects of nutritional factors, particularly vitamins and antioxidants, on oocyte quality in the context of gynecological cancers. The review aimed to summarize and synthesize existing research findings, identify gaps in the current evidence, and propose directions for future research.

### 2.2. Search Strategy

A comprehensive literature search was conducted in major databases, including PubMed, Scopus, Web of Science, and Google Scholar, covering publications up to October 2024. The following keywords and their combinations were used: “gynecological cancer”, “oocyte quality”, “fertility preservation”, “vitamin A”, “vitamin C”, “vitamin D3”, “vitamin E”, “Coenzyme Q10”, “antioxidants”, “oxidative stress”, and “ovarian reserve”. Boolean operators (AND, OR) were applied to optimize search results.

### 2.3. Inclusion and Exclusion Criteria

- Inclusion Criteria: Studies were included if they:

○ Investigated the impact of vitamins and antioxidants on oocyte quality.
○ Focused on patients with gynecological cancers (e.g., ovarian, cervical, and endometrial cancers).
○ Evaluated outcomes related to oxidative stress, mitochondrial function, or oocyte morphology.
○ Were original research articles, clinical trials, systematic reviews, or meta-analyses published in English.

- Exclusion Criteria: Studies were excluded if they:

○ Focused solely on male infertility or non-gynecological cancers.
○ Did not report specific outcomes related to oocyte quality.
○ Were conference abstracts, commentaries, or editorials without primary data.

### 2.4. Data Extraction and Synthesis

Relevant data were extracted from each included study, including information on study design, sample size, type of gynecological cancer, interventions (e.g., specific vitamins or antioxidants), and reported outcomes related to oocyte quality (e.g., oxidative stress markers, mitochondrial function, fertilization rates). A qualitative synthesis was performed to summarize the findings and identify consistent patterns and discrepancies across studies.

## 3. Molecular Aspects of Oogenesis, Oocyte Quality, and Gynecological Cancers

**Figure 1:**
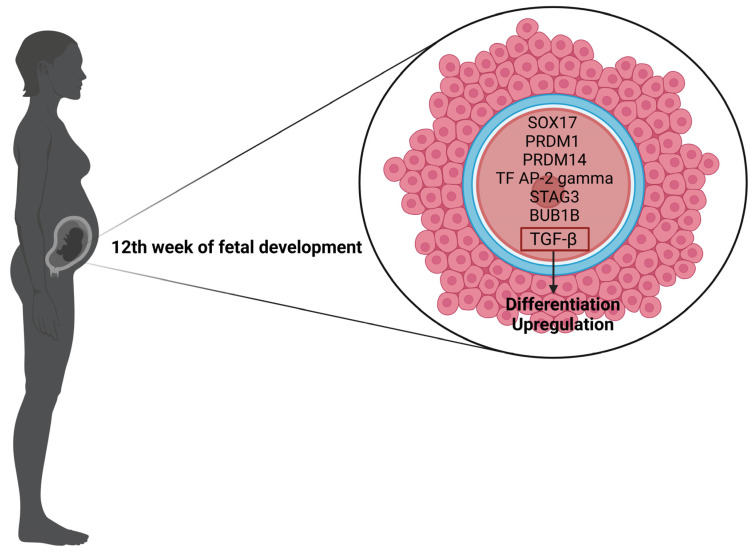
Molecular processes involved in oocyte development. Created in BioRender. Andone, B., https://BioRender.com/z57o718 (accessed on 6 December 2024).

Oogenesis is the process of oocyte formation at the ovarian follicle level. The process starts in fetal ovaries when primordial germ cells turn into oogonia in the 12th week of fetal development. Meiosis begins, triggering different steps, such as chromosome pairing and crossing over. Not only does meiosis play a key role in the development of oocytes, but also the interactions between the gamete and the granulosa cells and cumulus cells [7]. In these very early stages of gamete formation, several genes and transcription factors are important: SOX17, PRDM1, PRDM14, transcription factor AP-2 gamma, involved in upregulating germ cells and inhibiting somatic markers [8], STAG3, involved in chromosome segregation, and BUB1B, responsible for the formation of mitotic checkpoint serine/threonine kinase B [7], playing an important role in the kinetochore formation and dynamics [8]. After primary oocytes have developed from oogonia, they are arrested in prophase I (germinal vesicle stage), enclosed in primordial follicles, and remain dormant until puberty. As the hormonal status changes after puberty, the follicles undergo maturation, together with the oocyte, one follicle per cycle. Selected oocytes exit the dormant state, resume meiosis I, extrude the polar body, reach the metaphase of meiosis II, and develop into secondary oocytes, which are ovulated. Figure 1 emphasizes the main genes and molecular processes involved in oocyte development.

The success of fertilization depends on the quality and potency of the oocyte. The oocytes are encompassed in cumulus cells (CC), which are responsible for supporting all throughout the gamete development. Not only do the oocytes depend on the cumulus cells, but cumulus cell differentiation, regulation, and intercellular interaction depend on molecules from transforming growth factor-β (TGF-β) formed by the oocytes. The cumulus–oocyte complex (COC) integrity is sustained by a hyaluronic acid-rich extracellular matrix (ECM). Different studies showed that cumulus cells can influence the oocyte developmental capacity [9]. As oocyte provides the energy for embryo development, it has high amounts of energy resources, especially fatty acids and glucose. In order to trigger the glycolysis cascade, mature oocytes secrete paracrine factors that are able to upregulate glycolysis. Interestingly, studies show that removal of oocytes from the cumulus–oocyte complexes decreases the level of glycolytic enzymes [7].

The zona pellucida (ZP) is a glycoprotein coat at the interface between oocyte and surrounding cells, formed from four glycoproteins: ZP1, ZP2, ZP3, and ZP4, interacting with one another. The zona pellucida is essential in the fertilization process, as the spermatozoa attach to the ZP, binding specifically to a sialic acid molecule attached to the ZP3 molecule. The structure of the ZP can predict the fertilization potency of the oocyte. Mature oocytes, ready for fertilization, present a sponge-like structure of the ZP with a high density of pores, while a smooth-surfaced ZP is associated with immature oocytes [9]. hZP genes are responsible for creating the ZP protein complex. Studies reveal that mutations at the level of hZP genes are associated with infertility. For example, a homozygous deletion of 8 bp in hZP1 is associated with the absence of the ZP. In the same gene, a missense mutation leads to an abnormal ZP and degenerated oocytes. Gene variations in hZP2 and hZP3 are responsible for producing proteins that cannot undergo normal secretion and assembly during oocyte growth. This will lead to a decreased stability of the gap junction between oocytes and the follicle membrane, compromising the nutrition of oocytes [10].

The perivitelline space (PVS) is the space found between the plasma membrane of the oocyte and the zona pellucida. It contains a hyaluronan-rich extracellular matrix and the polar body. PVS size can affect the fertilization rate, polyspermy rate, and embryo quality [9]. Embryo quality refers to the developmental potential of an embryo, typically evaluated based on morphological grading, cell division rate, and blastocyst formation. High-quality embryos have a higher likelihood of implantation and successful pregnancy [11].

The polar body (PB) represents a small cellular byproduct of meiosis, extruded in PVS, between the ZP and the plasma membrane. The PB can predict the oocyte quality. An intact PB is associated with higher fertilization rates, and higher embryo quality. PB integrity can be affected by maternal age. Studies have shown that in advanced maternal age, the PB can be degenerated and deviated from the oocyte’s spindle [9].

The ooplasm represents the cytoplasmic compartment, represented especially by mitochondria, which are the most abundant, followed by the endoplasmic reticulum (ER) and Golgi bodies (GB). Cytoplasmic morphology can predict the competence of the oocyte. A light-colored cytoplasm is associated with low-density organelles and a low developmental potential [9,11,12]. Vacuole presence is described to be responsible, with a low yield of good-quality blastocysts, a high biochemical pregnancy rate, but low clinical pregnancy. Granulation in the ooplasm can predict a low yield of competent blastocysts, and a low pregnancy rate [12,13]. SER aggregates and different inclusions lead to a low fertilization rate, low embryo quality, and low pregnancy rate. The same outcomes have been reported also in the case of high-viscosity ooplasm [9,12].

The ultrastructure of the oocyte is modified in oncological syndromes. Fabiani et al. described that in cancer, the ovary response to stimulation treatment and oocyte quality decrease. They have described a significant difference between a cancer group and a control group, with a higher mean of immature oocytes and abnormal oocytes in the cancer group. The abnormalities included cytoplasmic dysmorphism, granules in the perivitelline space, the presence of vacuoles, and high-degree degradation of the ooplasm. This can be explained in the context of the presence of a continuous inflammatory microenvironment and increased oxidative stress, associated with tumoral development [4]. Alvarez et al. reported a decrease in the oocytes retrieved after ovarian stimulation in patients with gynecological cancers. Furthermore, the number of mature oocytes, fertilization rate, and embryo development were decreased in these patients compared with the control group and the patients with other types of malignancies [14]. In a novel bioinformatics study, Zheng et al. concluded that BTG4 mRNA expression is correlated with the aggressiveness of gynecological cancers, being negatively related to ovarian cancer survival. They have also reported that BTG4 is responsible for impaired oocyte and embryonic development, but further investigation of the relationship between mutant BTG4 in gynecological cancers and oocyte competency is required [15]. Not only is the oocyte ultrastructure directly affected by the microenvironment created by the cancerous cells, but the cumulus cells can also show dysregulations, leading to a decreased oocyte competency. Assou et al. demonstrated that in patients with ovarian and lung cancers, CCs show overexpression of the genes related to the HER2 signaling pathway: ERBB3, ERBB4, and PARD3, and also of the cell signaling and proliferation factors: MCM10, CCNE2, CDK6, MDM2, and CCND3. These overexpressions will lead to dysregulations in the oocyte development pathway, impairing it [16].

## 4. Antioxidants That Can Influence Oocyte Quality

### 4.1. Vitamin A

Retinoic acid, which is a metabolite of vitamin A, is associated with IVF success, oocyte quality, and cumulus cell complex development, as shown in a very recent study of Sidell and Rajakumar. Connexin-43 (Cx43), the main protein involved in gap junctions between cumulus granulosa cells (CGCs), is regulated by retinoic acid, increasing their number and improving the gap junction communication. A high number of gap junctions enhances the communication between the cumulus–oocyte complex, increasing oocyte competency. In this way, retinoic acid could be used to increase the quality of the COC, which is decreased in cancerous syndromes [17]. Kazemi et al. reported a positive correlation between the cleaved-embryo rate and vitamin A uptake before the start of the IVF cycle [18]. All-trans retinoid acid (ATRA) is another category of vitamin A metabolites. Even though it is not proven that they can increase the oocyte quality, it was demonstrated by Lokman et al. that it can inhibit the annexin A2-S100A10 signaling pathway, stopping ovarian cancer proliferation, indirectly increasing the reproductive capacity of the patient [19]. Table 1 shows the main effects of vitamin A in gynecological cancers, oocyte quality, and embryo quality.

### 4.2. Vitamin C

Vitamin C, also known as ascorbic acid, is a powerful antioxidant with a vital role in cellular health, particularly for patients undergoing cancer treatments [20]. Vitamin C may help preserve oocyte quality by mitigating oxidative stress and inflammation, two major factors that compromise ovarian function and fertility [21,22,23]. Its protective properties are especially relevant for patients undergoing chemotherapy and radiotherapy, as these treatments often lead to increased oxidative damage in ovarian tissues [24,25,26], negatively impacting oocyte viability and quality. One of the primary ways vitamin C supports oocyte quality is through its ability to scavenge free radicals and reduce oxidative damage in ovarian cells [23]. Studies have shown that high levels of reactive oxygen species (ROS) can impair mitochondrial function within oocytes, leading to poor energy production and cellular degradation [27,28,29,30]. Vitamin C’s antioxidant activity neutralizes these free radicals, preserving mitochondrial integrity and, consequently, enhancing oocyte viability. In a study of cancer patients receiving high-dose vitamin C alongside their treatment, the results showed reduced markers of oxidative stress in reproductive tissues [31], suggesting that vitamin C supplementation may shield oocytes from some of the damaging effects of cancer therapies [32,33].

In addition to reducing oxidative stress, vitamin C is essential for collagen synthesis, which is vital for maintaining the structural integrity of ovarian follicles [34,35]. Follicles with strong structural support are more likely to protect and sustain oocytes, leading to an enhanced quality during the maturation process. Research has also indicated that sufficient vitamin C levels are associated with a more balanced hormonal environment, which is crucial for proper ovarian function. Vitamin C aids in the regulation of estrogen and progesterone synthesis, both of which are essential for the development and maturation of oocytes [36]. By stabilizing hormone levels, vitamin C may help to create an environment conducive to optimal oocyte development [36,37,38].

Furthermore, vitamin C has been shown to modulate inflammatory pathways, reducing the presence of pro-inflammatory cytokines such as interleukin-6 (IL-6) and tumor necrosis factor-alpha (TNF-α). Elevated levels of these cytokines are commonly observed in gynecological cancers and can exacerbate follicular atresia (degeneration of follicles), which negatively affects oocyte quality and reserve [34]. By lowering cytokine activity, vitamin C can potentially support better oocyte morphology and improve fertilization potential in cancer patients.

Vitamin C supplementation may contribute to improved outcomes in assisted reproductive techniques (ART), such as in vitro fertilization (IVF), particularly for patients with compromised ovarian function due to cancer treatments. By preserving oocyte quality through antioxidant and anti-inflammatory effects, vitamin C may serve as a valuable adjunct in fertility preservation protocols. Although more research is needed to establish specific dosages and confirm its effects on long-term reproductive outcomes, current evidence underscores the potential role of vitamin C in enhancing oocyte quality and protecting fertility in gynecological cancer patients. Table 1 shows the main effects of vitamin C in gynecological cancers, oocyte quality, and embryo quality.

### 4.3. Vitamin D3

Vitamin D3, or cholecalciferol, is increasingly recognized in reproductive medicine for its influence on oocyte quality, especially in patients facing the challenges of gynecological cancers. Upon intake, vitamin D3 is converted into its active form, calcitriol, which then interacts with vitamin D receptors (VDRs) expressed in ovarian tissue, granulosa cells, and the endometrium [39]. This interaction is significant, as VDR activation plays a role in regulating genes associated with cell growth, inflammation, and steroid hormone synthesis, all of which are essential in maintaining oocyte quality and overall ovarian function [39,40].

Studies have shown that vitamin D3 may protect ovarian tissue from oxidative stress, which is a known factor in the degradation of oocyte quality, particularly in women undergoing chemotherapy and radiotherapy [41]. A recent study found that women with sufficient levels of vitamin D3 had higher ovarian reserve markers, including higher anti-Müllerian hormone (AMH) levels and antral follicle count (AFC), compared to those deficient in vitamin D [42]. These results, although promising, are controversial, as some other studies suggest no correlation between vitamin D intake and AMH [43], whereas others suggest a beneficial role of vitamin D in infertility, but just in lower doses [44], and need to be further investigated on other populations and higher cohorts. As cancer treatments often diminish these markers, vitamin D3 may counteract some of the adverse effects on fertility commonly experienced by cancer patients.

Additionally, vitamin D3’s anti-inflammatory properties help mitigate inflammatory cytokines that are elevated in gynecological cancers and can disrupt the delicate ovarian environment. Calcitriol has been observed to reduce TNF-α, IL-6, IL-4 [45], IL-1β [46] and inhibit the NF-κB [47] signaling pathway, which are linked to follicular degeneration and poor oocyte outcomes [48]. This reduction in inflammatory response is particularly beneficial for cancer patients, as they are associated with better oocyte morphology and increased fertilization rates.

Vitamin D3 also supports endocrine regulation within the ovaries by enhancing sensitivity to follicle-stimulating hormone (FSH) and luteinizing hormone (LH), both essential for follicular development and oocyte maturation. Increased FSH sensitivity is linked to improved follicular growth and maturation, which may lead to better-quality oocytes capable of successful fertilization [49]. Research further indicates that optimal vitamin D3 levels are correlated with improved mitochondrial function in oocytes, which is critical for energy production and cellular integrity during fertilization [49].

For gynecological cancer patients, vitamin D3 supplementation could thus serve as a targeted adjunct therapy to maintain oocyte quality, reduce oxidative damage, and potentially improve reproductive outcomes. While ongoing studies aim to clarify the precise mechanisms and optimal dosages, the current evidence suggests that maintaining sufficient levels of vitamin D3 may provide a protective effect against the reproductive side effects of cancer treatments, thereby enhancing fertility preservation options for these patients. Table 1 shows the main effects of vitamin D3 in gynecological cancers, oocyte quality, and embryo quality.

### 4.4. Vitamin E

Vitamin E, a vital fat-soluble antioxidant [49], has gained attention for its protective role in preserving oocyte quality, particularly in gynecological cancer patients who face increased oxidative stress and inflammation due to cancer treatments. Composed mainly of tocopherols and tocotrienols, with alpha-tocopherol as the most biologically active form [50], vitamin E’s potent antioxidant properties help protect ovarian cells from the damage induced by chemotherapy, radiotherapy, and the inflammatory microenvironment made by cancerous cells, which are known to reduce oocyte quality and overall ovarian reserve. Besides this, vitamin E has been demonstrated to show a protective effect against developing gynecological cancer [51].

Oocytes are particularly susceptible to oxidative damage because they contain a high density of mitochondria and lipid-rich membranes, making them prime targets for lipid peroxidation by free radicals. Vitamin E’s antioxidant action stabilizes cell membranes by donating hydrogen atoms to neutralize free radicals, thus preventing the peroxidation of lipids and safeguarding the integrity of oocyte membranes. This protection is crucial, as oxidative damage to mitochondria impairs ATP production, which is essential for the energy-intensive processes of oocyte maturation, fertilization, and early embryonic development. Studies have demonstrated that vitamin E supplementation reduces oxidative stress markers in ovarian tissue, preserving mitochondrial function and energy availability within oocytes, ultimately enhancing their viability and developmental potential [52,53,54,55,56]. An interesting recent study of Faisal et al. reported that in patients undergoing IVF, the highest ratio of mature oocytes and grade I embryos was obtained when the blood levels of alpha-tocopherol were 10–15 mg/L, whereas at higher concentrations of a-tocopherol, the MtII oocyte yield and grade I embryos decreased, emphasizing that vitamin E concentration is a crucial aspect that needs to be taken into consideration [57].

Beyond its role as an antioxidant, vitamin E also modulates inflammatory pathways, which is critical in the context of gynecological cancers. Cancer treatments often induce chronic inflammation, elevating pro-inflammatory cytokines such as tumor necrosis TNF-α and IL-1β. These cytokines are associated with accelerated follicular atresia and can negatively impact oocyte quality by degrading the ovarian environment. By reducing TNF-α and IL-1β levels, vitamin E lowers the inflammatory burden on ovarian tissue, helping to preserve the structural integrity of follicles [58,59,60]. Animal studies indicate that vitamin E supplementation reduces ovarian inflammation and improves follicular survival rates, providing a more supportive environment for oocyte development. This anti-inflammatory effect also protects granulosa cells, which play a crucial role in nurturing oocytes and facilitating nutrient transfer, further enhancing oocyte quality [61].

In addition to its antioxidant and anti-inflammatory effects, vitamin E supports hormonal balance, which is essential for proper oocyte maturation. Vitamin E has been shown to improve the sensitivity of ovarian cells to follicle-stimulating hormone (FSH), a critical hormone for follicular growth and development. Cancer treatments often disrupt hormonal balance, negatively impacting folliculogenesis and oocyte quality. By enhancing FSH sensitivity, vitamin E helps ensure that follicles receive adequate stimulation for maturation, which is fundamental for producing high-quality oocytes. Additionally, vitamin E stabilizes estrogen levels, promoting the proliferation of granulosa cells and supporting a healthy ovarian environment conducive to oocyte maturation. This hormonal regulation is particularly beneficial for cancer patients who often face hormonal imbalances due to treatment, which can further compromise fertility [62,63].

Vitamin E’s potential benefits extend to ART, such as IVF, where it has shown promise in improving outcomes for patients with compromised ovarian function. In a study involving women undergoing IVF, those who received vitamin E supplementation exhibited higher-quality embryos and increased fertilization rates [54]. These improvements are likely due to the protective effects of vitamin E on oocyte health and the reduction in oxidative stress within follicular fluid, which is crucial for successful fertilization. Additionally, vitamin E has been observed to enhance luteal phase support by reducing oxidative stress in the corpus luteum, which produces progesterone essential for sustaining early pregnancy.

As promising as these findings are, the determination of optimal vitamin E dosages for gynecological cancer patients remains an area for further research. While moderate doses have shown protective effects, excessive intake of vitamin E, being a fat-soluble vitamin, may lead to adverse effects. Future studies are needed to establish safe and effective dosage guidelines that maximize its benefits for fertility preservation without risking toxicity. Table 1 shows the main effects of vitamin E in gynecological cancers, oocyte quality, and embryo quality.

### 4.5. Coenzyme Q10

Coenzyme Q10 (CoQ10) is a lipid-soluble electron transporter essential for the stability of mitochondrial complex III, being a major cellular antioxidant. It is also essential in maintaining the membrane potential, being involved in ATP formation. A CoQ10 deficiency leads to mitochondrial dysfunctions. Bentov et al. reported that in a group treated with CoQ10 2 months prior to IVF, the quality of the embryos increased, and the rate of aneuploidy decreased, suggesting that CoQ10 was able to improve the mitochondrial function and offer protection against oxidative stress [64].

Another interesting study by Xu et al. demonstrates that the supplementation of CoQ10 before starting the IVF cycle decreases the period of stimulation and increases the number of oocytes retrieved, the fertilization rate, and the number of high-quality embryos. ROS, which can also result from cancer, induce DNA damage in oocytes, leading to mutations and apoptosis. CoQ10 shows the capacity of ameliorating these effects. Also, by improving mitochondrial function, CoQ10 is associated with increasing the quality of oocytes, an increased fertilization rate, an a higher yield of good-quality embryos [65].

In older oocytes a diminished expression of Pdss2 and Coq6, enzymes responsible for CoQ10 synthesis, was observed. In these oocytes, Ben-Meir et al. reported reduced ATP production and increased meiotic spindle abnormalities, leading to infertility. In Pdss2-knockout model animals, the ovarian reserve was diminished, leading to premature ovarian failure [66]. The same authors showed in another study that CoQ10 might improve cumulus cell quantity and quality, improving the quality of the oocytes. CoQ10 can recover the mitochondrial distribution and function in oocytes.

Mitochondrial dynamics is an important aspect of oocyte competency. Zhang et al. showed in their study that CoQ10 can decrease abnormal mitochondria distribution and dynamics in aged oocytes by up to 35%. They also showed that CoQ10 supplementation suppresses ROS-induced DNA damage and apoptosis [67]. Varela and Labarta reported similar results in a study in 2021. They stated that supplementation of CoQ10 prior to IVF treatments increases the number of cumulus cells, leading to increased oocyte competency [68]. These studies emphasize the antioxidant effect of CoQ10, necessary in an inflammatory microenvironment specific to gynecological cancers. In order to increase the chances of IVF success, oocyte quality should be high, and CoQ10 supplementation can increase it. Table 1 shows the main effects of coenzyme Q10 on gynecological cancers, oocyte quality, and embryo quality.

**Figure 2:**
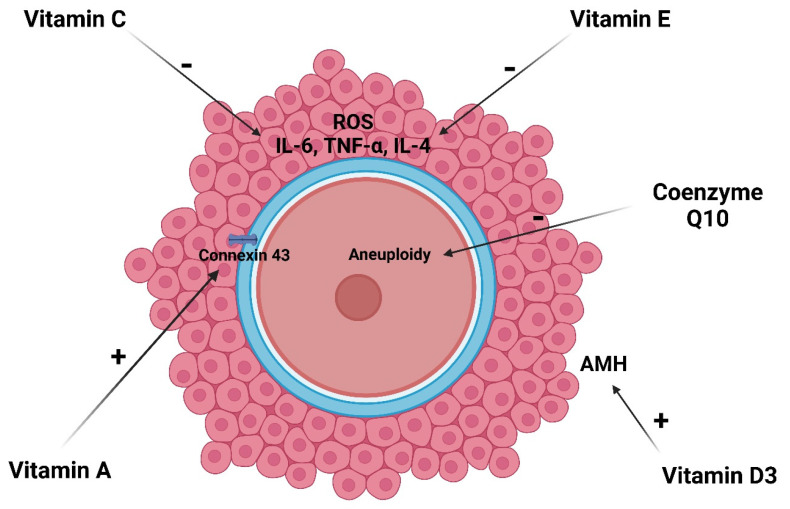
Effect of vitamins on the oocyte quality and reproductive capacity of gynecological cancer patients. Created in BioRender. Andone, B., https://BioRender.com/i52f443 (accessed on 11 November 2024).

Figure 2 shows the main effects of antioxidants on oocyte quality and fertility capacity of gynecological cancer patients.

**Table 1:** Main effects of vitamins on gynecological cancers, oocytes quality, and embryo quality.

Antioxidant	Effect on Oocyte Quality	Effect on Embryo Development	Ref
Vitamin A	Stabilizes and increases the quality of the cumulus–oocyte complex Regulates connexin 43 in granulosa cells, increasing oocyte competency	Increases the quality and the number of cleved-stage embryos	[17,18,69,70,71,72]
Vitamin C	Higher yield of MtII oocytes	Higher number of good-quality embryos	[20,23,73]
Vitamin E	Higher yield of MtII oocytes Protects against ROS effects in COV434 granulosa cells in gynecological cancer chemotherapy, increasing oocyte utilization in fertility preservation	Higher number of good-quality embryos Culture media enhanced with vitamin E increased the blastocyst development rate	[53,73,74,75,76]
Vitamin D3	Increases AMH	No effect on blastulation rate	[42,77,78]
Coenzyme Q10	Increases ovarian response Increases oocyte quality	Increases embryo development rate	[65,79,80]

## 5. Conclusions

In conclusion, gynecological cancers present significant challenges to both survival and reproductive health, particularly by affecting oocyte quality. The mutations associated with cancer and the effects of treatments like chemotherapy and radiotherapy contribute to oxidative stress, inflammation, and other cellular disruptions, impairing the developmental potential of oocytes. To address these issues, fertility preservation techniques such as oocyte cryopreservation are essential, yet their success hinges on the initial quality of the oocytes. Emerging research underscores the importance of certain vitamins and antioxidants, which offer promising avenues for improving oocyte quality in gynecological cancer patients. Vitamins A, C, D3, and E, along with coenzyme Q10, have demonstrated potential in mitigating the effects of oxidative stress and inflammation, improving mitochondrial function, and stabilizing hormonal balance, all of which are crucial for oocyte health. By enhancing the structural and metabolic resilience of oocytes, these substances may serve as valuable adjuncts in fertility preservation strategies. In addition, antioxidants could provide broader benefits by protecting ovarian function during gonadotoxic treatments, potentially preserving natural fertility in cancer survivors. This approach aligns with improving the overall quality of life for women who wish to conceive post-treatment, assuming successful cancer remission. While further studies are required to determine optimal dosages and treatment protocols, these findings offer hope for improving reproductive outcomes for women facing the dual burden of cancer and fertility impairment.
